# Correlates of reported modern contraceptive use among postpartum HIV-positive women in rural Nigeria: an analysis from the MoMent prospective cohort study

**DOI:** 10.1186/s12978-018-0663-8

**Published:** 2019-01-08

**Authors:** Eric E. Chinaeke, Chinenye Fan-Osuala, Miriam Bathnna, Chamberline E. Ozigbu, Babayemi Olakunde, Habib O. Ramadhani, Echezona E. Ezeanolue, Nadia A. Sam-Agudu

**Affiliations:** 1 Department of Clinical Pharmacy and Outcome Sciences, University of South Carolina College of Pharmacy, Columbia, USA; 2 International Research Center of Excellence, Institute of Human Virology Nigeria, Abuja, Nigeria; 3 Department of Health Services Policy and Management, Arnold School of Public Health, University of South Carolina, Columbia, USA; 4 School of Community Health Sciences, University of Nevada Las Vegas, Las Vegas, NV USA; 5 National Agency for the Control of AIDS, Abuja, Nigeria; 6 Division of Epidemiology and Prevention, Institute of Human Virology, University of Maryland School of Medicine, Baltimore, USA; 7 Healthy Sunrise Foundation, Las Vegas, USA; 8 Department of Paediatrics and Child Health, University of Nigeria, Enugu, Nigeria

**Keywords:** Contraception behavior, Contraceptive agents, HIV, PMTCT, Rural populations, Nigeria

## Abstract

**Background:**

Nigeria has an annual population of ~ 200,000 women who are both pregnant and HIV-positive. High unmet need for family planning in this population could lead to unintended pregnancies, along with the increased risk of mother-to-child transmission of HIV (MTCT). To identify modifiable barriers and facilitators in effective family planning, we examined correlates of modern contraceptive use among HIV-positive women enrolled in the MoMent prevention of MTCT (PMTCT) implementation research study in rural North-Central Nigeria.

**Methods:**

In this prospective cohort study, HIV-positive pregnant women were enrolled at 20 Primary Healthcare Centers and followed up to 12 months postpartum. Baseline socio-demographic, clinical and obstetric data were collected at enrollment. Participants were to receive routine family planning counselling from healthcare workers during postnatal visits. Analysis utilized baseline data linked to available family planning information collected from each woman at the first postpartum visit. Multivariate logistic regression was performed to determine factors associated with modern contraceptive use.

**Results:**

Out of 497 women enrolled, family planning data was available for 399 (80.3%) women, of whom 349 (87.5%) received family planning counselling, and 321 (80.5%) were 30 years old or less. Two-thirds (268, 67.2%) of the cohort analyzed had 1–2 children at baseline; 24.8% (*n* = 99) had 3–4 children, and 8.0% (*n* = 32) had > 4 children. Approximately half (199, 49.9%) of the women reported no modern contraceptive use in the postpartum period. Male condoms (116, 29.1%) were the most reported method of contraception; other methods reported included oral hormones (71, 17.8%) and intrauterine devices (13, 3.2%). Only disclosure of HIV status to male partner or relative (aOR = 2.0, 95% CI: 1.2–3.3; *p* = 0.01) and receipt of family planning counselling (aOR = 2.3, 95% CI: 1.1–4.8; *p* = 0.03) were positively associated with reported modern contraceptive use. Age, marital or educational status, religious affiliation, employment status, gravidity and parity were non-correlates.

**Conclusions:**

Family planning counselling and disclosure of HIV status are modifiable positive predictors of contraceptive use among our cohort of postpartum HIV-positive women in rural Nigeria. Rates of unintended pregnancy and concomitant risk of MTCT could be significantly reduced through strategies that facilitate these correlates.

**Clinical trials registration:**

Clinicaltrials.gov registration number: NCT 01936753; registered September 3, 2013.

## Plain English summary

Family planning is important in the prevention of HIV from mothers to infants. Unplanned pregnancies among women living with HIV are more likely to result in HIV-positive babies. Nigeria has nearly 200,000 pregnant HIV-positive women delivering every year. Poor access to and use of family planning methods among these women may result in unplanned pregnancy and increased infant HIV infections. Identifying factors that influence contraceptive use can guide policy and interventions for infant HIV prevention. We assessed for factors influencing contraceptive use among 399 HIV-positive women in rural North-Central Nigeria who were newly-delivered mothers. Only 50% of these women reported using contraceptives, with condoms being the most frequently used, followed by oral contraceptive pills and lastly, intra-uterine devices. We further found that women who received family planning counselling or disclosed their HIV status to male partners or others were more likely to use contraception. Our results support the strengthening of interventions for increasing access to family planning and improving rates of disclosure among women living with HIV, especially in rural Nigeria.

## Background

In 2017, there were an estimated 3.1 million people living with HIV in Nigeria, with women comprising approximately 55% of this population [1]. Nigeria has an annual population of nearly 200,000 HIV-positive pregnant women, representing the second-largest prevention of mother-to-child transmission of HIV (PMTCT) burden globally [2, 3]. There is also a high unmet need for family planning among Nigerian women in general, with only 10.8% of married women using any form of modern contraception [4]. The female-predominance in Nigeria’s HIV epidemic, large PMTCT burden and high unmet need for family planning demonstrate the need to include effective modern contraception as a component of comprehensive HIV care to women of reproductive age (MTCT) [5–7]. Additionally, Nigeria’s adoption of lifelong antiretroviral therapy (ART) for PMTCT (Option B+) in 2016 [8] creates further urgency for providing effective contraception for increasing numbers of women on HIV treatment who wish to avoid unintended pregnancies.

MTCT still accounts for the majority of new child HIV infections worldwide; this applies to the estimated 36,000 Nigerian children newly infected with HIV in 2017, the highest globally [9]. Nigeria’s PMTCT efforts have focused largely on increasing uptake and coverage of maternal ART [3], however, family planning counseling and provision of acceptable agents to address the contraceptive needs of women living with HIV are also paramount for successful PMTCT. For women living with HIV, family planning has the potential to delay motherhood and reduce unwanted pregnancy-consequently reducing maternal morbidity/mortality, improving child spacing and empowering them to discontinue child-bearing on reaching their desired family size [10]. Prevention of unintended pregnancies among HIV-infected women through family planning is cost-effective and could avert more new child HIV infections compared to perinatal transmission averted with antiretroviral drugs alone [10–14]. To this end, the World Health Organization (WHO) has long-recommended quality family planning as part of comprehensive PMTCT service delivery [15].

Despite the benefits and recommendations for contraception among women living with HIV, there have been historical challenges to successful implementation in Nigeria. Overall, access to, and utilization of modern contraception has been low in Nigeria, largely due to structural and individual-level barriers [16]. The Nigerian healthcare system lacks robust budgetary allocations for family planning, often leaving healthcare facilities with shortages in contraception commodities [17]. Utilization of modern contraceptives such as hormonal agents among Nigerian women is as low as 10%, and nearly 30% of women interested in family planning do not have access to contraceptives [4, 16, 17]. Furthermore, according to previous studies, knowledge of contraception among Nigerian women living with HIV is high, however, level of knowledge is not commensurate with level of contraceptive use, and this appeared to be associated with high numbers of unintended pregnancies [17, 18].

While prior studies have reported low contraceptive use among Nigerian women living with HIV, the predictors of utilization or lack thereof-remain unclear. This evidence is particularly important to generate in rural areas, where utilization of maternal-child health services-including PMTCT and family planning- is especially low [19–21]. This study aims to identify key predictors of contraceptive use among post-partum women living with HIV in high HIV-burden rural communities in North-Central Nigeria.

## Methods

### Study design and setting

This article presents cross-sectional data on family planning collected as part of the larger MoMent Nigeria prospective cohort study. Details of the prospective study’s design, setting and site selection have been previously published [22]. Briefly, the MoMent study evaluated the impact of structured (supervised activities, standardized training and reporting tools) peer support on maternal PMTCT retention and infant presentation for early HIV diagnosis [23, 24]. It was conducted in rural and semi-rural areas of the Federal Capital Territory and Nasarawa state, located in Nigeria’s North-Central region. At 5.8%, the prevalence of HIV among pregnant women in this zone ranks highest in the country [25]. The North-Central zone also has the highest prevalence of HIV among the general population in rural areas, at 3.7% [25]. As of 2013, knowledge of contraceptive use in this region was 78.6% while contraceptive use was among the lowest in the country at 15.6% [20].

The MoMent study was implemented at 20 primary healthcare centers (PHCs) across the two study states. Using criteria based on duration of PMTCT service provision, ANC clinic flow, numbers of HIV-positive women identified, staff strength, and EID service availability, the 20 study sites were selected from 102 available PHCs and assigned to intervention or control arm as matched pairs [22]. These 20 PHCs were located in 20 different towns and villages spanning nine local government areas (districts) [26], and were each staffed by 4 to 6 PMTCT providers comprising largely nurse-midwives and community health extension workers.

The intervention was structured peer support, which consisted of formal baseline training, a detailed scope of work, supportive supervision by a designated supervisor, a standardized logbook for recording client interactions, periodic performance audits of peer counsellors as well as a systematic client tracking process [22]. The intervention package was compared to routine, less-supervised, less structured peer support. All study participants were followed up from enrollment up to 12 months postpartum and received routine Option B PMTCT care according to the prevailing national guidelines [27]. All participants were also expected to receive family planning counselling from healthcare workers during routine postnatal visits.

### Recruitment procedures

Since this was a sub-study within the larger prospective MoMent study, the sample available for this analysis was limited to the sample recruited for the MoMent study overall. Details of the sample size calculations have been published in the protocol paper [22]. Ultimately, 497 women were recruited in the prospective study as follows: HIV-positive pregnant women were recruited from antenatal care (ANC) clinics of study PHCs to receive either structured support or routine peer support. Eligible participants were aged 15 years or more, had made a minimum of one ANC visit before delivery and planned to continue receiving antenatal and postnatal care at their PHC of recruitment [22]. Both ART-naïve and ART-experienced pregnant women (irrespective of gestational age) were recruited; those presenting in labor were excluded. HIV screening was conducted routinely for all pregnant women presenting to ANC clinics at the study sites, and women who were new or known HIV-positive were subsequently linked to a Mentor Mother (intervention sites) or routine peer counsellor (control sites) by a healthcare worker. Thereafter, study staff would approach women interested in participating in the study for informed consent. Recruitment was conducted between April 2014 and September 2015.

### Data collection

Standardized case report forms were used to collect baseline and family planning data of study participants by face-to-face interviews and abstraction from medical records. Information on participants’ socio-demographic, clinical and obstetric history including age, marital status, level of education, religion, employment status, parity, time of HIV diagnosis, and HIV disclosure status were collected at enrollment. As part of routine study data collection, family planning information was collected during clinic visits made by each woman within the 12-month postpartum period. Participants were asked about their contraceptive of choice and whether they received postpartum family planning counselling from their healthcare provider.

All responses were based on self-report, and no verification of contraceptive use was conducted. Data analysis was restricted to the first postpartum clinic visit where family planning data was available for collection. All study data was obtained by study staff directly from participants and/or from facility maternal-child registers.

### Data analysis

Frequency and distribution of contraceptive use among study participants was described using simple proportions. The outcome of interest for the correlation analysis was modern contraceptive use which was defined as the self-reported use of one of the following modern contraceptive methods: condoms, intrauterine devices, and hormonal contraceptives, with an “other” category reserved for less-used methods such as sterilization. Reports of traditional methods such as abstinence and no method of contraception were categorized as “no modern contraceptive use”. These categorizations were based on definitions from the United Nations Population Division [4].

To assess for correlates of contraceptive use, participants’ baseline socio-demographic and obstetric data were linked to their postpartum family planning data. Analysis was restricted to women with available family planning information. Multivariate logistic regression was performed to test for variables independently associated with contraceptive use. Covariates assessed included socio-demographics (age, marital status, religion), socio-economic factors (employment status, level of education), obstetrics (parity, gravidity, contraceptive choice, receipt of family planning) and HIV care (HIV disclosure status, timing of HIV diagnosis).

For women missing family planning data due to death, loss-to-follow-up or non-response, a sensitivity analysis was performed using baseline demographic and obstetric characteristics for imputation. Variables included in the multiple imputation model were socio-demographics (age, marital status, religion), socio-economic factors (employment status, level of education), obstetrics (parity, gravidity, contraceptive choice, receipt of family planning) and HIV care (HIV disclosure status, timing of HIV diagnosis).

Results of the multivariate analysis were presented as adjusted odds ratios (aORs) with *p*-values and confidence intervals (CI). Statistical significance was considered at *p* < 0.05 and for confidence intervals excluding the value “1”. The analysis was performed using STATA version 12 (Statacorp, College Station, Texas).

### Ethical considerations

The study was approved by the Nigerian National Health Research Ethics Committee, the Ethics Review Committee of the World Health Organization and the Institutional Review Board of the University of Maryland, Baltimore. All study participants provided written informed consent. All approvals allowed for the inclusion of participants 15 to less than 18 years of age as they were considered able to provide written informed consent for research participation due to their status as pregnant women. Safeguards against intense community-level stigma for participants included contractual requirements for Mentor Mothers to maintain client confidentiality especially with regard to HIV status, the option to arrange client visits at alternate locations if home visits were not acceptable, and use of generically-labeled Mentor Mother logbooks that did not include the term “HIV.”

## Results

### Baseline antenatal characteristics of enrolled women

A total of 497 HIV-positive pregnant women were enrolled in the MoMent study. Two women died and 52 were lost to follow-up before delivery, leaving 443 women alive and available in the postpartum period. Of these 443 women, 399 (90.1%) had available data on receipt or non-receipt of postpartum family planning counselling and contraceptive choice. Nearly 90% (349/399) of these women received family planning counselling in the postpartum period. At the time of antenatal enrollment, 321 (80.5%) of the 399 women were aged 30 years or less, 380 (95.2%) were married, and 203 (50.9%) had received secondary or higher-level education.

### Postpartum status of surveyed HIV-positive women

Data on the post-delivery time-points at which participants were first surveyed on postpartum contraceptive use were collated. A total of 353 (88.5%) women with family planning data available were surveyed between 0 and 3 months postpartum; 24 (6.0%) were > 3 to 6 months postpartum; 11 (2.8%) were > 6 to 9 months postpartum, and 8 (2.0%) were > 9 to 12 months postpartum; this included 5 women who were surveyed between 1 and 3 weeks beyond their 12-month postpartum date.

### Reported postpartum use of modern contraceptives

Of the 399 women with available data, approximately half (49.9%, 199) reported no contraceptive use. Among the remaining half (*n* = 200), male condoms were the most frequently reported method of contraception (116, 58.0%), followed by oral hormonal contraception (71, 35.5%) and intra-uterine devices (13, 6.5%) (Table 1). There were no reports of the use of injectables, female condoms or male or female sterilization for contraception.

**Table 1:** Reported Modern Contraceptive Use among Postpartum Women Living with HIV

Contraceptive Type	All Women (*N* = 399) n (%)
Male condoms	116 (29.1)
Oral Hormones	71 (17.8)
Intra-uterine devices	13 (3.2)
Injectable Hormones	0 (0.0)
Female condoms	0 (0.0)
Male/female sterilization	0 (0.0)
None	199 (49.9)

### Correlates of reported postpartum modern contraceptive use

Multivariate analysis showed that women who had disclosed their HIV status to a male partner or relative (aOR = 2.0, 95% CI: 1.2–3.3; *p* = 0.01) or who had received family planning counselling (aOR = 2.3, 95% CI: 1.1–4.8; *p* = 0.03) were more likely to have reported using contraceptives than those who had not (Table 2). Parity of > 4 trended towards, but did not achieve significance as a correlate of reported contraceptive use. Interestingly, women exposed to structured Mentor Mother support were less likely to report contraceptive use (aOR = 0.5, 95% CI: 0.3–0.8, *p* < 0.01). There were no associations between modern contraceptive use and the remaining variables evaluated-including education, religious affiliation or employment status.

**Table 2:** Correlates of Reported Modern Contraceptive Use among Women Living with HIV

Characteristics	All Women *N* = 399	Contraceptive Use n (%)^a^	aOR (95% CI)	*P* value
Type of Peer Support				
Routine Peer Support	153 (38.4)	91 (59.5)	1.0	
Mentor Mother	246 (61.7)	109 (44.3)	**0.5 (0.3–0.8)**	**< 0.01**
Age, years				
< 21	39 (9.7)	14 (35.9)	1.0	
21–30	282 (70.7)	148 (52.5)	1.5 (0.6–4.0)	0.37
> 30	78 (19.6)	38 (48.7)	1.5 (0.7–3.2)	0.34
Marital status				
Married	380 (95.5)	191 (50.3)	1.0	
Single^b^	18 (4.5)	9 (50.0)	1.1 (0.4–3.1)	0.83
Educational level attained				
None	106 (26.6)	57 (53.8)	1.0	
Primary	90 (22.5)	39 (43.3)	0.8 (0.4–1.4)	0.41
Secondary	164 (41.1)	83 (50.6)	0.9 (0.5–1.6)	0.71
Tertiary	39 (9.8)	21 (53.9)	0.9 (0.4–2.2)	0.81
Religious affiliation				
Christian	270 (67.7)	132 (48.9)	1.0	
Muslim	129 (32.3)	68 (52.7)	1.1 (0.7–1.8)	0.75
Employment status				
Employed – skilled	58 (14.5)	27 (46.6)	1.0	
Employed – unskilled	61 (15.3)	39 (63.9)	1.9 (0.9–4.2)	0.09
Unemployed	280 (70.2)	134 (47.9)	1.0 (0.5–1.9)	0.93
HIV disclosure status^c^				
No	101 (25.3)	37 (36.6)	1.0	
Yes	298 (74.7)	163 (54.7)	**2.0 (1.2–3.3)**	**0.01**
Time of HIV diagnosis				
Previously diagnosed	166 (41.6)	95 (57.2)	1.0	
Newly diagnosed	233 (58.4)	105 (45.1)	0.8 (0.5–1.3)	0.31
Parity				
0–2	268 (67.2)	135 (50.4)	1.0	
3–4	99 (24.8)	54 (54.6)	0.9 (0.5–1.6)	0.71
> 4	32 (8.0)	11 (34.4)	0.3 (0.1–1.0)	0.05
Gravidity				
1	76 (19.1)	34 (44.7)	1.0	
2–4	250 (62.7)	132 (52.8)	1.2 (0.4–3.5)	0.70
> 4	73 (18.3)	34 (46.6)	1.1 (0.6–2.0)	0.87
Counselled on family planning				
No	46 (11.7)	15 (32.6)	1.0	
Yes	349 (88.4)	181 (51.9)	**2.3 (1.1–4.8)**	**0.03**

aOR, adjusted odds ratio; CI, confidence interval

p value < 0.05 = statistically significant. Bolded aORs, CIs and *p* values denote statistically significant results

^a^Denotes row percentage of women reporting contraceptive use

^b^Includes single, widowed, divorced and separated women

^c^Disclosure to male partner or relative

Due to the significant amount (98/497, 19.7%) of missing family planning data, a sensitivity analysis was performed to verify the robustness of the results (Table 3). Here, exposure to Mentor Mother support lost statistical significance while disclosure status (aOR = 2.1, CI: 1.3–3.3; *p* < 0.01) and receipt of family planning counselling (aOR = 3.1, CI: 1.8–5.3; p < 0.01) retained significance.

**Table 3:** Correlates of Reported Modern Contraceptive Use among Women Living with HIV: Sensitivity Analysis

Characteristics	All Women *N* = 497	Contraceptive Use n (%)^a^	aOR (95% CI)	P value
Type of Peer Support				
Routine Peer Support	237 (47.7)	101 (42.6)	1.0	
Mentor Mother	260 (52.3)	110 (42.3)	0.7 (0.5–1.1)	0.14
Age, years				
< 21	54 (10.9)	15 (27.8)	1.0	
21–30	352 (70.8)	156 (44.3)	1.5 (0.8–3.1)	0.23
> 30	91 (18.3)	40 (44.0)	1.8 (0.8–4.3)	0.18
Marital status				
Married	472 (95.0)	202 (42.8)	1.0	
Single^b^	25 (5.0)	9 (36.0)	0.6 (0.3–1.6)	0.37
Educational level attained				
None	146 (29.4)	60 (41.1)	1.0	
Primary	106 (21.3)	42 (39.6)	0.8 (0.5–1.5)	0.54
Secondary	194 (39.0)	86 (44.3)	0.8 (0.5–1.4)	0.53
Tertiary	51 (10.3)	23 (45.1)	0.8 (0.4–1.6)	0.47
Religious affiliation				
Christian	319 (64.2)	137 (43.0)	1.0	
Muslim	178 (35.8)	74 (41.6)	1.0 (0.6–1.5)	0.93
Employment status				
Employed – skilled	71 (14.3)	29 (40.9)	1.0	
Employed – unskilled	68 (13.7)	40 (58.8)	1.9 (0.9–3.8)	0.09
Unemployed	258 (72.0)	142 (39.7)	1.1 (0.6–2.0)	0.74
HIV disclosure status^c^				
No	129 (26.0)	38 (29.5)	1.0	
Yes	368 (74.0)	173 (47.0)	**2.1 (1.3–3.3)**	**< 0.01**
Time of HIV Diagnosis				
Previously diagnosed	197 (39.6)	101 (51.3)	1.0	
Newly diagnosed	300 (60.4)	110 (36.7)	0.7 (0.5–1.1)	0.13
Parity				
0–2	333 (67.0)	144 (43.2)	1.0	
3–4	122 (24.6)	55 (45.8)	1.0 (0.6–1.7)	0.94
> 4	42 (8.4)	12 (28.6)	0.5 (0.2–1.3)	0.15
Gravidity				
1	95 (19.1)	37 (39.0)	1.0	
2–4	303 (61.0)	139 (45.9)	0.9 (0.5–1.7)	0.85
> 4	99 (19.9)	35 (35.4)	0.7 (0.3–1.8)	0.46
Counselled on family planning				
No	107 (21.5)	25 (23.4)	1.0	
Yes	390 (78.5)	186 (47.9)	**3.1 (1.8–5.3)**	**< 0.01**

aOR, adjusted odds ratio; CI, confidence interval

*p* value < 0.05 = statistically significant. Bolded aORs, CIs and *p* values denote statistically significant results

^a^Denotes row percentage of women reporting contraceptive use

^b^Includes single, widowed, divorced and separated women

^c^Disclosure to male partner or relative

We further analyzed separately for correlates of the most frequently reported contraceptives: male condoms and oral hormones. Table 4 shows that when condom use was compared to no contraception, exposure to Mentor Mother support was associated with decreased odds of reported use (aOR = 0.2, CI: 0.1–0.4; p < 0.01). Conversely, women who had disclosed had higher odds of reported condom use d (aOR = 4.9, CI: 2.3–10.6; *p* < 0.01). For oral hormones versus none, women with more than 4 children had lower odds of reported use (aOR = 0.2, CI: 0.0–0.9; *p* = 0.04). Family planning counselling was not associated with reported use of either type of contraception when analyzed separately.

**Table 4:** Correlates of Reported Types of Modern Contraception Used among Women Living with HIV: Male Condoms and Oral Hormones vs No Contraception

Characteristics	Male Condoms aOR (95% CI)	P value	Oral Hormones aOR (95% CI)	P value
Type of Peer Support				
Routine Peer Support	1.0		1.0	
Mentor Mother	**0.2 (0.1–0.4)**	**< 0.01**	1.2 (0.6–2.5)	0.53
Age, years				
< 21	1.0		1.0	
21–30	2.4 (0.7–8.5)	0.17	0.8 (0.2–3.2)	0.80
> 30	1.9 (0.7–5.6)	0.23	1.1 (0.4–3.1)	0.93
Marital status				
Married	1.0		1.0	
Single^a^	1.2 (0.3–4.3)	0.79	1.6 (0.4–5.9)	0.47
Educational level attained				
None	1.0		1.0	
Primary	0.6 (0.3–1.6)	0.34	0.8 (0.3–1.9)	0.57
Secondary	1.1 (0.5–2.4)	0.74	0.9 (0.4–2.0)	0.71
Tertiary	1.8 (0.6–4.9)	0.27	0.3 (0.1–1.4)	0.12
Religious affiliation				
Christian	1.0		1.0	
Muslim	0.6 (0.3–1.4)	0.24	1.1 (0.7–1.8)	0.75
Employment status				
Employed – skilled	1.0		1.0	
Employed – unskilled	2.0 (0.7–5.4)	0.17	2.3 (0.9–6.1)	0.10
Unemployed	1.2 (0.5–2.8)	0.68	0.9 (0.4–2.1)	0.81
HIV disclosure status^b^				
No	1.0		1.0	
Yes	**4.9 (2.3–10.6)**	**< 0.01**	1.0 (0.5–1.9)	0.97
Time of HIV Diagnosis				
Previously diagnosed	1.0		1.0	
Newly diagnosed	1.0 (0.5–1.8)	0.91	0.7 (0.3–1.4)	0.28
Parity				
0–2	1.0		1.0	
3–4	1.0 (0.5–2.1)	0.57	0.9 (0.4–2.1)	0.71
> 4	0.7 (0.2–2.8)	0.96	**0.2 (0.0–0.9)**	**0.04**
Gravidity				
1	1.0		1.0	
2–4	0.7 (0.2–2.7)	0.65	2.2 (0.6–8.3)	0.26
> 4	0.9 (0.4–2.1)	0.82	0.9 (0.4–2.2)	0.85
Counselled on family planning				
No	1.0		1.0	
Yes	1.8 (0.7–4.6)	0.20	2.7 (0.8–8.6)	0.11

aOR, adjusted odds ratio; CI, confidence interval

*p* value < 0.05 = statistically significant. Bolded aORs, CIs and *p* values denote statistically significant results

^a^Includes single, widowed, divorced and separated women

^b^Disclosure to male partner or relative

## Discussion

Our study reports a high unmet need for family planning among postpartum women living with HIV in rural Nigeria. Results show that during the postpartum period, half of these women did not use modern contraceptives. Where modern contraception was used postpartum, male condoms had the highest uptake in our population. Receipt of family planning counselling and disclosure of HIV status were significantly associated with contraceptive use.

Our results are consistent with in-country studies, which have reported low modern contraception utilization among women living with HIV in Nigeria [17, 18]. Oyebode et al. reported that 87% of post-partum HIV-positive women in Jos, North-Central Nigeria confirmed not using any female-targeted contraception, only 37% always used male condoms, and only 38% had planned their last pregnancy [17]. From both urban and rural clinics in Zaria, North-West Nigeria, Shehu et al. reported that only 36% of post-partum HIV-positive women interviewed were using contraception, with male condoms being the most commonly-used (60%), followed by injectables (22%), oral hormones (10%), female sterilization (2.3%), and implants and intra-uterine devices at 1.1% each [18]. The predominance of reported male condom use among women in our study and others is expected, despite their lower failure rates in the prevention of unintended pregnancies [28]. Unfortunately, the uptake of more effective contraceptive methods such as male/female sterilization, implants, and intra-uterine devices is limited by multiple factors including poor knowledge, myths, misconception and fear of side effects [29, 30]. However, unlike condoms, these methods are unable to prevent sexually-transmitted infections [28]. Even when women desire consistent condom use, gender power imbalances may affect their ability to negotiate with their partners [31–33]. The practical and socio-cultural challenges of condom use (whether alone or as dual protection) among HIV-affected couples need to be further explored, with the development of counter-strategies in mind.

Our findings also corroborate with other studies conducted in similar settings in sub-Saharan Africa, which have reported high unmet need for family planning and low modern contraceptive use among HIV-infected women in rural areas, and enumerate factors that may promote uptake of family planning in this population [34, 35]. In these studies, unmet need for family planning ranged between 34 and 48% [34, 35]. Poor knowledge, fear of adverse effects, lack of male partner support, unavailability and inaccessibility to contraceptive commodities or services, and cultural norms hinder the use of effective contraception among HIV-infected women [35–37].

Our study indicates that modern contraceptive use is significantly associated with receipt of family planning counselling among women living with HIV. Family planning counselling is a modifiable factor that can improve contraceptive use, which in turn may lead to significant reductions in unintended pregnancy and MTCT among women living with HIV. Our results are consistent with prior studies in other parts of sub-Saharan Africa [35, 38–41], which report that family planning counselling delivered by trained healthcare workers, especially where integrated with HIV services, improves modern contraceptive use by providing correct information and educating clients on different methods and their safety, modes of action, and effectiveness. This is important in rural areas, where there is often limited access to correct family planning information and effective contraceptive commodities [42, 43]. Interventions that focus on efficient and acceptable delivery of comprehensive family planning counselling are needed, especially in high HIV-burden rural settings where health systems are relatively weaker and missed opportunities for PMTCT may be more likely. Delivery of these services could be expanded and sustained through trained community workers or lay peer mentors to overcome the relative lack of professional healthcare workers and inadequate counselling skills that often limit provision of family planning counselling to HIV-infected women [42, 44]. Of note, structured peer support provided by Mentor Mothers emerged as a negative correlate of reported condom use when compared with no contraception. This is unexpected and may be due to sample size limitations; neither type of peer support was associated with reported contraceptive use in the overall sensitivity analysis. MoMent’s Mentor Mother training curriculum did not focus on contraception or family planning counselling and this may explain why there is no appreciable effect.

Disclosure of HIV status to male partners and relatives was also associated with uptake of contraception in our study. Disclosure is also a modifiable factor, and this finding reflects the key role male partners play in family planning decision-making, particularly in Nigeria [45, 46]. Studies from other parts of sub-Saharan Africa have also reported lower odds of modern contraception uptake among HIV-positive women whose partners were not aware of the women’s HIV status [47–49]. Despite increasing evidence on the association between HIV status disclosure and positive PMTCT outcomes [50–52], only two-thirds of HIV-positive African women disclose to their male partners [53]. Reasons for non-disclosure in this population include avoiding blame, divorce, neglect, or domestic violence [54, 55]. Recognizing the significance of disclosure and its potential impact, the WHO has recommended that assisted partner notification services be offered as part of HIV testing and care services [56]. Partner notification services and the facilitation of couples’ HIV counselling and testing have been shown to improve partner disclosure [56, 57].

Our results further indicate that educational level, religious affiliation and employment status did not correlate with modern contraceptive use in our cohort. These findings contradict results from similar studies among HIV-positive women in other African countries [35, 38, 39]. Our findings also contradict previous studies conducted among Nigerian women in the general population. For instance, Adebowale et al. reported that married women who were more educated/earned more than their husbands or were employed were more likely to use modern contraception, whereas Muslim women were less likely to do so [58]. Ejembi et al. identified level of education as a positive predictor of modern contraceptive use among Nigerian women; Muslim religious affiliation was also a negative correlate [59].

It should be noted that our study population differs from the comparators, in that ours was a sample of < 500 HIV-positive women in rural communities, whereas the other Nigerian reports were nationwide Demographic and Health Surveys among tens of thousands of women across both rural and urban communities. Our smaller sample size and narrow geographical scope may have limited our ability to identify religion, employment and/or education as correlates of modern contraceptive use. Within our cohort, educational status and Christian religious affiliation were positively associated with facility delivery [23], however, not for post-partum modern contraceptive use. The role of HIV-positive and/or rural status with regard to education, religion and employment as correlates of modern contraceptive use in Nigeria is unclear. This needs to be further explored. The paucity of relevant published data from our local study setting makes it challenging to assess our findings in the context of prior evidence. Of note, one study conducted among ~ 400 HIV-positive women in Christian-dominant South-East Nigeria also reported non-correlation of education (primary vs post-primary) and religion (Catholic vs Non-Catholic) with the use of modern contraception [60].

Regardless of contradictions with some prior studies, our study has identified two modifiable factors-receipt of family planning counselling and partner HIV status disclosure-that can lead to an increase in contraceptive use among women living with HIV. Developing feasible, culturally-appropriate and sustainable strategies to deliver these interventions in rural areas will be key to closing PMTCT gaps in Nigeria and potentially, in similar high HIV-burden, limited-resource settings.

### Study limitations

Our study has some limitations. Given that our data were based on respondents’ self-reports, our findings may be subject to recall bias and/or invalid responses, leading to an over- or under-estimation of actual contraceptive use. This may have contributed to some of the contradictory findings to prior evidence previously discussed. We did not assess respondent’s prior receipt of family planning counselling or use of contraception, which ultimately may have confounded our estimations. Lastly, we did not evaluate for changes in reported contraceptive use over an extended time during the postpartum period.

## Conclusions

Family planning counselling and disclosure of HIV status were associated with uptake of modern contraception in our cohort of women in rural Nigeria. These are modifiable factors that are amenable to strengthening and scale-up at both facility and community level. In rural areas where human capital may be limited and health systems are relatively weak, task-shifting contraception and disclosure counselling to trained community health workers and laypersons may potentially facilitate expansion and sustainability of these services. Innovative, culturally-appropriate yet gender-empowered approaches to improve male partner disclosure and meet unmet needs for family planning among women living with HIV remain critical to improving PMTCT outcomes in high HIV-burden, resource-limited settings.

## Abbreviations

AIDS: Acquired Immune Deficiency Syndrome
ANC: Antenatal Care
ART: Antiretroviral Therapy
HIV: Human Immunodeficiency Virus
MoMent: Mother Mentor
MTCT: Mother-To-Child Transmission of HIV
PHC: Primary Healthcare Center
PMTCT: Prevention of Mother-To-Child Transmission of HIV
WHO: World Health Organization
